# Compression Characteristics and Fracture Simulation of Gluten Pellet

**DOI:** 10.3390/foods12081598

**Published:** 2023-04-10

**Authors:** Zongyou Ben, Abdulaziz Nuhu Jibril, Xiao Sun, Yu Bai, Duoxing Yang, Kunjie Chen, Yan Dong

**Affiliations:** 1 College of Engineering, Nanjing Agricultural University, Nanjing 210031, China; 2 College of Biotechnology and Food Engineering, Chuzhou University, Chuzhou 239000, China; 3 Anhui Bi Lv Chun Biotechnology Co., Ltd., Chuzhou 239200, China

**Keywords:** gluten pellet, moisture content, elastic modulus, compressive strength, failure energy, fracture simulation

## Abstract

Gluten pellets are readily broken on packaging and transportation. This study aimed to research mechanical properties (elastic modulus, compressive strength, failure energy) with different moisture contents and aspect ratios under different compressive directions. The mechanical properties were examined with a texture analyzer. The results revealed that the material properties of the gluten pellet are anisotropic, and it was more likely to cause crushing during radial compression. The mechanical properties were positively correlated with the moisture content. The aspect ratio had no significant effect (*p* > 0.05) on the compressive strength. The statistical function model (*p* < 0.01; R^2^ ≥ 0.774) for mechanical properties and moisture content fitted well with the test data. The minimum elastic modulus, compressive strength, and failure energy of standards-compliant pellets (with moisture content less than 12.5% d.b.) were 340.65 MPa, 6.25 MPa, and 64.77 mJ, respectively. Moreover, a finite element model with cohesive elements was established using Abaqus software (Version 2020, Dassault Systèmes, Paris, France) to simulate the compression rupture form of gluten pellets. The relative error of the fracture stress in the axial and radial directions between the simulation results and the experimental value was within 4–7%.

## 1. Introduction

Feed is essential in aquaculture, accounting for 60 to 70 percent of the total cost. The demand for feed is rising in tandem with the fast growth of aquaculture [1]. Pellet feed is one of the most popular types of feed due to its comprehensive nutrition, outstanding palatability, and ease of storage and transportation. After pelleting, the pellet feed must go through conveying, bagging, storage, and transportation [2]. External forces such as collision and compression will necessarily be present in these processes. Protein, with a level of 75–85%, is an essential component of the gluten pellet [3]. The pellets may be broken under the above loads, leading to increased powder content and decreased processing quality. Ultimately, it will have a more substantial impact on feed production enterprises’ economic benefits.

Most scholars focus on the factors that affect the performance of pellets. The feed’s composition is complex and significantly affects cohesiveness, resilience, springiness, hardness, transverse crushing load, and mechanical durability [4,5]. The mechanical damage of agricultural products will increase broken rates, which is closely related to their mechanical properties. The asymptotic modulus of biomass pellets increased linearly with an increase in compressive pressure (1000, 2000, 3000, 4000, and 4400 N), and particle sizes (3.2, 1.6, and 0.8 mm) of wheat straw did not produce any significant difference on pellet density [6]. The tensile strength of wheat straw pellets was improved with values ranging from 1.13 to 1.63 MPa with added binders (crude glycerol, bentonite, lignosulfonate, and wood residue) [7,8]. Diametral tensile strength, axial compressive strength, and bulk modulus of ground biomass pellets in the early stages of densification (<100 kPa) were determined, and the vital quality metrics of switchgrass pellets can be predicted using bulk modulus [9]. Moisture content is another critical factor [10,11]. He et al. [12] investigated the compressive force of freshly hulled lotus seeds, finding that the maximum compressive force was connected to the loading method, grade, loading rate, and resting period when the grain was deformed. Shu et al. [13] investigated the damage characteristics of amylose to various rice cultivars using compression and three-point bending tests. Kruszelnicka [14] and Babic et al. [15] investigated the link between corn’s mechanical characteristics, secant elastic modulus, and fracture energy under static compression.

With the advancement of finite element theory, it is now possible to simulate the mechanical properties of agricultural goods under actual load using a computer [16]. Peng et al. [17] used the discrete element approach to model and simulate pig feed and investigate pellet feed’s chewiness and crushing behavior during chewing. Celik et al. [18] performed a compression test on pecans and used the nonlinear finite element approach to simulate the high-speed crushing of hickory, revealing the hickory crushing mechanism. The finite element method was utilized by Ashtiani et al. [19] to estimate the mechanical damage of grapefruit under the influence of an external compressive force. The Hertz-Mindlin contact model, based on the discrete element approach, was used by Romuli et al. [20] to simulate the shelling of Jatropha curcas fruit. The results reveal that the highest shelling efficiency is 98.8%, with a 477 kg/h feed rate. However, scholars assume that the material properties of the research object are isotropic in the simulation. There are few literatures reports at home and abroad on the related research on the mechanical properties and anisotropic compression crushing simulation of gluten pellet feed.

Hence, this work takes gluten pellets as the research object and aims to investigate the effect of compressive direction, moisture content, and aspect ratio on elastic modulus, compressive strength, and failure energy. Meanwhile, the rupture form was simulated by Abaqus software (Version 2020, Dassault Systèmes, Paris, France) with anisotropic material properties. The results of this study can provide technical parameters for the design and optimization of machinery in the production, processing, and transportation of gluten pellets and a guide for reducing the breakage rate of gluten pellets. It also provides a new train of thought for the anisotropy simulation of gluten pellets.

## 2. Materials and Methods

### 2.1. Sample Treatment and Measure Methods

Bilvchun Biotechnology Co., Ltd. (Chuzhou, Anhui, China) provided the gluten pellet material and produced it in Lai’an County, Chuzhou City, Anhui Province. The pellet was pale yellow, slender, and cylindrical, with 8.85% d.b. initial moisture content.

#### 2.1.1. Test Instrument

The test equipment is a TA.XT.plus type texture analyzer (Stable Micro System, Surrey, UK), the test force range is ±30 kg, the test force accuracy is 0.0002%, the test distance accuracy is 0.001 mm, and the test speed range is 0.01–40 mm/s. GZX-9240 MBE type electric heating blast drying oven (Shanghai Boxun Industrial Co., Ltd., Medical Equipment Factory, Shanghai, China). JA2003B type electronic balance (Shanghai Yueping Scientific Instrument Co., Ltd., Shanghai, China). DL91150 type electronic digital vernier caliper (Shanghai Yuanmai Trading Co., Ltd., Shanghai, China), measuring range 0–150 mm, accuracy ± 0.03 mm, resolution 0.01 mm.

#### 2.1.2. Test Sample Preparation

**Figure 1:**
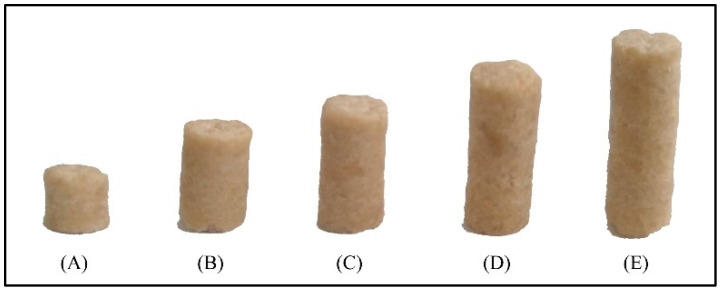
Compression test sample of the gluten pellet feed. (**A**–**E**) means aspect ratio is 1, 1.5, 2, 2.5, and 3, respectively.

To obtain samples with an aspect ratio of 1, 1.5, 2, 2.5, and 3, as shown in Figure 1, 360-mesh water-resistant sandpaper and digital vernier caliper were used to smooth both ends of the pellets and measure diameter and height, respectively. The average diameter of the samples was 4.2 mm.

The method of evenly spraying a certain quality of deionized water on pellets was used to obtain pellet samples with different high moisture content to study the effect of moisture content on the compressive characteristics of pellets [21]. The initial moisture of the gluten pellet sample was 8.85% d.b., which was measured by direct drying method with a drying temperature of 103 ± 2 °C until the difference between the two weighings of the sample was less than 0.1% of its mass [22]. In addition, five parts of gluten pellets with initial moisture were weighed and placed in a dry dish with good tightness. The quality of deionized water required to prepare samples with different moisture contents was calculated by Equation (1).

(1) $$m=m_{0}\frac{H-H_{0}}{100-H}$$

where *m* is the mass of deionized water, g; *m*_0_ is the mass of the test sample, g; *H*_0_ is the initial moisture content of the test sample, %; and *H* is the target moisture content of the test sample, %.

The beaker contained deionized water attached to the heating sheet, and the single-layer tiled pellet was placed in a sealed incubator simultaneously. The pellets gradually absorbed the steam generated by the heated deionized water, and it was kept for 24 h to make the absorbed water uniform. Before the test, the pellets were taken out from the incubator, and the actual moisture content of the sample was measured. Each sample was tested five times, and the average value was taken as the sample’s moisture content. One part of the gluten pellet was treated with the direct drying method to reduce the moisture content. Finally, the moisture contents of the five groups of samples were 6.74%, 8.85%, 10.37%, 11.91%, and 14.53% d.b., respectively.

#### 2.1.3. Compression Mechanical Properties Test

**Figure 2:**
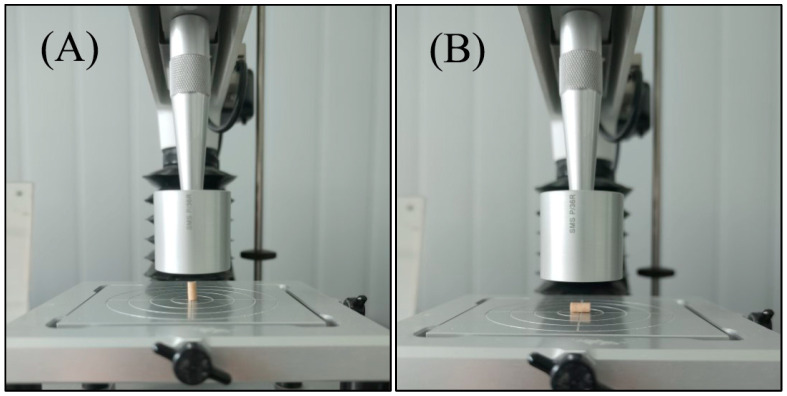
Compression test device of the gluten pellet feed. (**A**,**B**) means compressive direction is axial and radial, respectively.

The mechanical test of the pellet in its natural state is of great significance. The pellet is mainly subjected to compression loads under natural accumulation conditions such as transportation and storage. Due to the pellet’s regular shape, the pellet’s uniaxial compression mechanical properties along the axial and radial directions were tested in this paper. During the test, the samples were placed upright and flat on a square rigid base according to the axial and radial directions. A P/36R cylindrical indenter with a diameter of 36 mm was selected, as shown in Figure 2. The test speed was 0.5 mm/s, the trigger force was 5 g, the maximum compression distance was half the pellet’s height, and the device was preheated 30 min before testing.

When the indenter touches the pellet sample, it moves downward at the set speed. The device collected and recorded the displacement of the indenter and the corresponding force to generate a force-displacement curve. When the force on the curve rose to the maximum value and suddenly dropped, it meant that the sample had been fractured, and the test ended. Ten samples with the same aspect ratios of each moisture content were randomly selected for the compression test, and the average value was taken as the test result.

### 2.2. Sample Treatment and Measure Methods

In order to study the compressive strength of the pellet and its rupture form (the location and shape of the fracture cross-section) under compression load, Abaqus finite element analysis software (Version 2020, Dassault Systèmes, Paris, France) was used to globally insert cohesive elements into the pellet model with the Maxs damage criterion and combined with the anisotropic properties of pellets to analyze different moisture content pellet to contrast the compression test results. The compression simulation assumptions: the internal stress of the pellet is zero before compression; the temperature and moisture content of the pellet does not change during the compression process; and the tiny gaps between the particles in the pellet are ignored. According to statistics, the most frequent pellets are about 6.5–9 mm long. Therefore, the article selected a pellet with an aspect ratio of 2 to solve its finite element analysis model.

#### 2.2.1. Cohesive Zone Model

**Figure 3:**
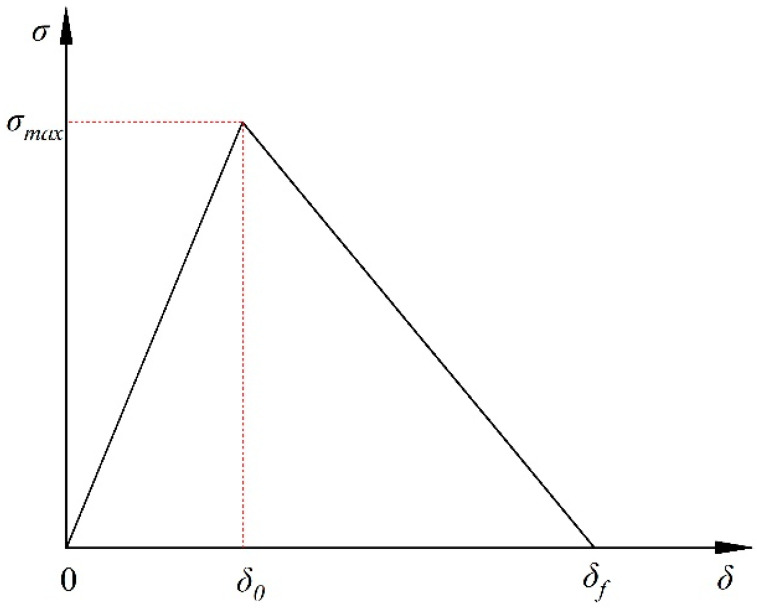
Bilinear cohesive model.

The cohesive zone model (CZM) was proposed by Dugdale [23] and is mainly used in crack propagation. Based on this, Mi et al. [24] developed the bilinear cohesive zone model by studying brittle fracture, as shown in Figure 3. It described the linear elastic phase and the softening phase of linear decrease in stiffness before and after the material reached the strength limit, respectively. The cohesion model was implemented in finite element by introducing the cohesion element, using the cohesion element to establish the relationship between the materials around the interface, and using the traction separation law (TLS) to define the cohesion damage in the form of tension displacement.

The initial damage criterion was calculated according to the Equation (2).

(2) $$max\left\{\frac{\langle \sigma_{n}\rangle }{\sigma_{n}^{0}},\frac{\sigma_{s}}{\sigma_{s}^{0}},\frac{\sigma_{t}}{\sigma_{t}^{0}}\right\}=1$$

where $\sigma_{n}$, $\sigma_{s}$, $\sigma_{t}$ are normal and tangential stress and $\sigma_{n}^{0}$, $\sigma_{s}^{0}$, $\sigma_{t}^{0}$ are normal and tangential named stress.

When the initial damage occurred to the material, it would enter the damage evolution stage. Displacement-based failure criterion refers to taking the ultimate opening displacement of the structural material as the basis for judging its complete failure. It could be expressed as Equation (3).

(3) $$\delta =\sqrt{{\langle \delta_{n}\rangle }^{2}+\delta_{s}^{2}+\delta_{t}^{2}}$$

where *δ* is crack opening displacement and $\delta_{n}$, $\delta_{s}$, $\delta_{t}$ are normal and tangential displacement.

#### 2.2.2. Physical Model and Parameter Setting

- Geometry and meshing

**Figure 4:**
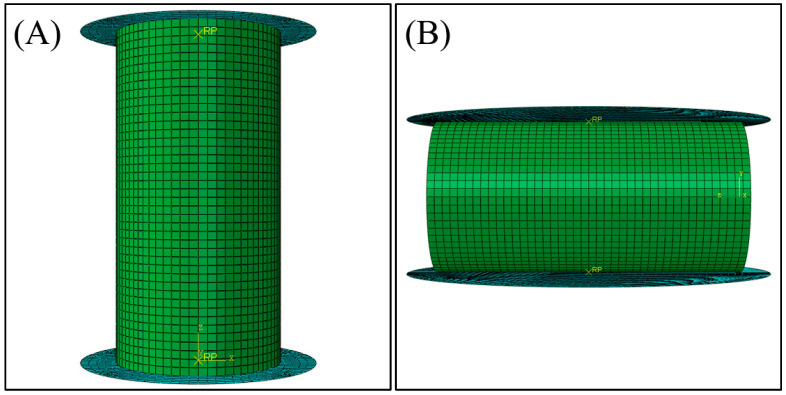
Geometric model and meshing of feed compression. (**A**,**B**) means compressive direction is axial and radial, respectively.

In order to simulate the actual compression process, a thin shell in contact with the pellet was created in the upper and lower positions of the compression direction as the base and the indenter, respectively, and set as a discrete rigid body. The pellet was set as a deformable body. In order to effectively express the law of crack generation and expansion, the mesh size of the pellet should be as small as possible. In this paper, the global mesh size was set to 0.2 mm, the base and the indenter were divided by free mesh, the element type was R3D4, the pellet was divided by sweep to generate hexahedral mesh, and the cohesive element type was COH3D8. The geometric model and meshing of the pellet are shown in Figure 4.

- Model parameters and boundary conditions

The model parameters were divided into base material and cohesive element parameters. The base material was a pellet, and its anisotropic physical property parameters were the density, which was obtained by calculation method, and the total volume, which was calculated according to the number of pellets with different aspect ratios. The total mass of the sample was weighed. The Poisson’s ratio of the three planes was the same [25]; the elastic modulus and shear modulus were the data measured by the experiment. They were inputted as engineering constants in the x, y, and z directions and XY, XZ, and YZ planes, respectively. Cohesive element parameters included bond strength, rigidity, and density [26]. The damage evolution adopted the displacement criterion. The specific physical parameters are shown in Table 1.

The pellet adopted a linear elastic stress-strain model. The base was a fixed constraint, the indenter was a speed constraint, and the compressive speed was 0.5 mm/s. General contact was adopted, and the ABAQUS/Explicit solver was used to solve it.

### 2.3. Statistical Analysis

SPSS (Version 25.0, International Business Machines Corporation, Armonk, NY, USA) and Origin (Version 2021b, OriginLab, Northampton, MA, USA) statistical software were used for data processing and analysis. Significance analysis was performed by analysis of variance (ANOVA), and the independence test was performed by *t*-test. Meanwhile, the significance level was set at *p* ≤ 0.05.

The compression test of pellet samples could measure the force-displacement curve of the sample. In order to obtain mechanical parameters such as elastic modulus, compressive strength, and failure energy of pellets, it was necessary to convert the relationship between force and displacement into the relationship between stress and strain.

#### 2.3.1. Elastic Modulus

The elastic modulus is the slope of the elastic compression stage on the stress–strain curve, determined by Equations (4) and (5).

(4) $$E_{A}=\frac{\sigma_{A}}{∆L/L}$$

(5) $$E_{R}=\frac{\sigma_{R}}{∆d/d}$$

where $E_{A}$, $E_{R}$ are axial and radial elastic modulus, MPa; $\sigma_{A}$, $\sigma_{R}$ are the axial and radial stress, MPa; ∆L, ∆d are the axial and radial deformation increment of the pellet feed, mm; and L, d are the length and diameter of the pellet feed, mm.

#### 2.3.2. Compressive Strength

The compressive strength is the maximum stress of the test sample when it breaks under the action of compressive load, which is determined by Equations (6) and (7).

(6) $$\sigma_{A}=\frac{4F_{max}}{\pi d^{2}}$$

(7) $$\sigma_{R}=\frac{2F_{max}}{\pi dL}$$

where $\sigma_{A}$, $\sigma_{R}$ are the axial and radial stress, MPa; $F_{max}$ is the maximum compressive failure force, N; and L, d are the length and diameter of the pellet feed, mm.

#### 2.3.3. Failure Energy

The compressive failure energy is the area under the first peak of the force-displacement curve. It was used to judge how much energy material absorbed.

## 3. Results and Discussion

### 3.1. Compression Properties of Pelleted Feed

**Figure 5:**
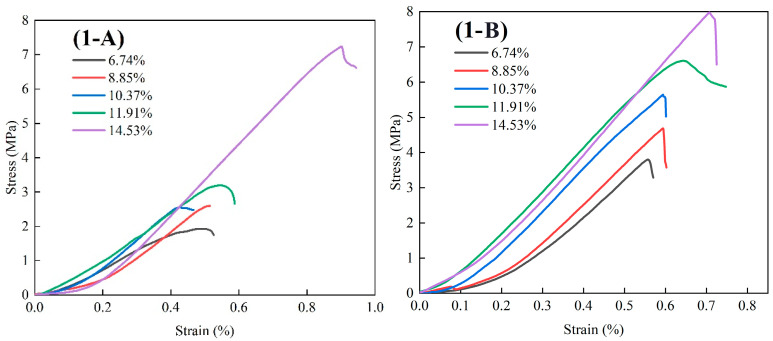

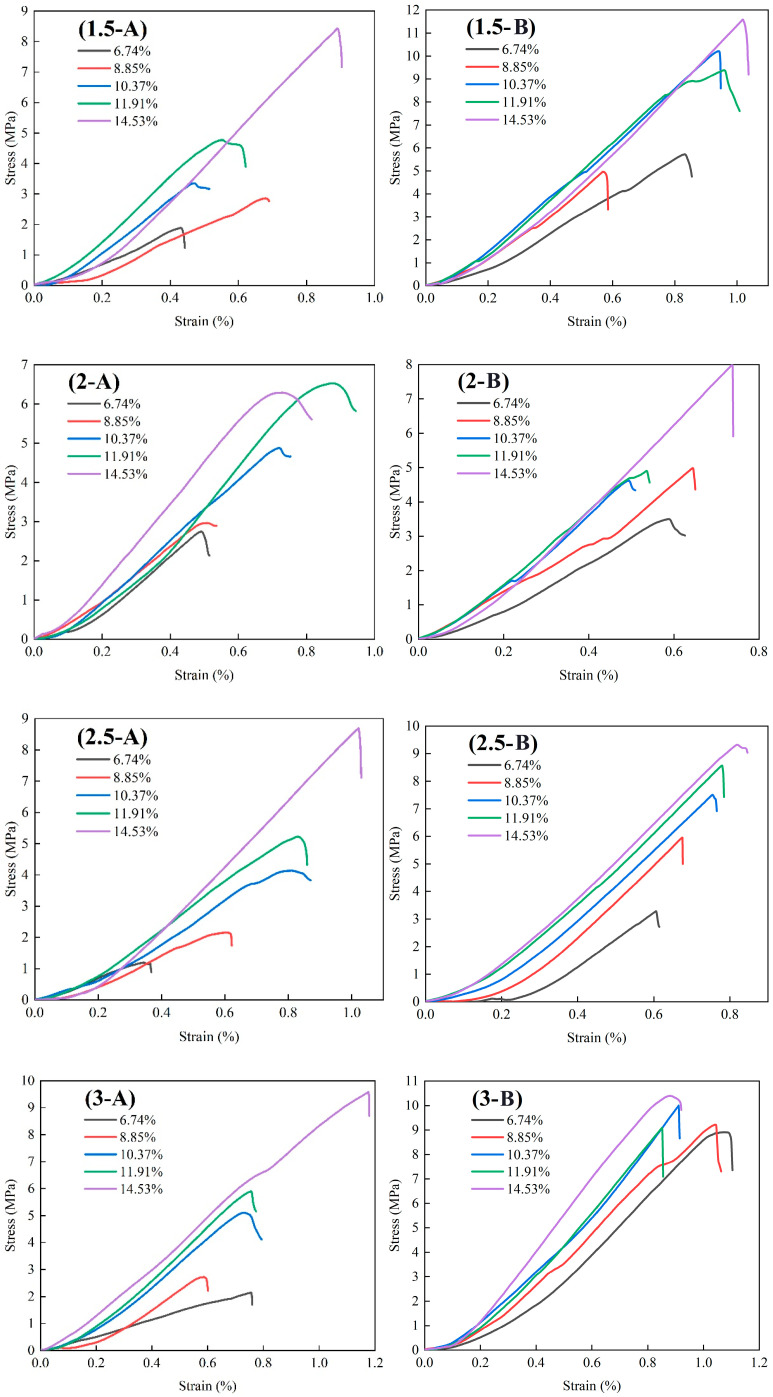
Compressive stress-strain curve of the pellet feed. The number 1, 1.5, 2, 2.5 and 3 before (**A**) or (**B**) means aspect ratio; (**A**,**B**) means compressive direction is axial and radial, respectively.

The force-displacement data collected and converted stress-strain data points in the test were imported into the Origin software (Version 2021b, OriginLab, Northampton, MA, USA), then plotted into the stress-strain curve with different moisture contents under compression, as shown in Figure 5.

Figure 5A shows the stress-strain curve change of the pellets’ axial compression with different aspect ratios and moisture content. The stress of the sample increased with the strain. The stress reached its maximum when the strain increased to a specific value. As the strain increased, the stress suddenly decreased, and the sample ruptured. The point corresponding to the maximum stress was the failure point of the pellets, and the stress was called the failure stress, that was, the compressive strength of the pellets. When the moisture content was higher than 11.91% d.b., the stress-strain curve would increase sharply, some curves would drop sharply at the failure stress point, and some curves would drop slowly, indicating that when different pellets broke, some broke suddenly and some broke gradually. With the increased moisture content, the stress and strain also increased when the pellets failed. The compressive stress–strain curve was curvilinear when the strain was less than 0.2% and linear when the strain was greater than 0.2%. It may be because the pellets feed was extruded from powder, and there were tiny gaps between the powders. It may also be likely due to flattening the rough surface of the pellets that needed to be polished flat. After the indenter contacted the pellet, the pellet went through three processes: compression, elastic deformation, and crushing. Figure 5B shows the variation of radial compression stress-strain curves of pellets with different aspect ratios with moisture content, which is generally similar to the law of axial compression.

Pellets’ elastic modulus, compressive strength, and failure energy were calculated for quantitatively expressing according to the stress-strain and force-displacement curve. If the slope of the stress-strain curve changed significantly and there was no apparent straight-line segment, the slope of the line connecting the origin of the coordinates on the stress-strain curve and the point corresponding to 1/3 of the ultimate stress on the curve, that is, the secant modulus could be taken as the elastic modulus of the pellets [27]. The elastic modulus, compressive strength, and failure energy of the gluten pellets with different aspect ratios, moisture contents, and compressive directions are shown in Table 2 after data processing.

The independence test of the elastic modulus of each pellet with different aspect ratios and moisture content under axial and radial compression was taken. It could be found that there was a significant difference (*p* < 0.05). Moreover, the radial value was significantly more extensive than the axial direction, as shown in Table 3. It indicated that the ability of the pellets to resist deformation in the axial and radial directions was different during the compression process, and the gluten pellets showed apparent anisotropy [28].

It can be seen from Table 4 that at the level of *α* = 0.05, the moisture content and the compressive direction had significant effects on the elastic modulus, compressive strength, and failure energy. At the same time, the aspect ratio had a significant effect on the elastic modulus and failure energy but had no significant effect on the compressive strength, which was inconsistent with the research results of Zhang et al. [29] that the aspect ratio has no significant effect on elastic modulus, compressive strength, and failure energy. Referring to the F-value, it could be seen that the main factors affecting the elastic modulus, compressive strength, and failure energy were the compressive direction, moisture content, and aspect ratio order.

### 3.2. Influence of Moisture Content and Aspect Ratio on the Elastic Modulus

**Figure 6:**
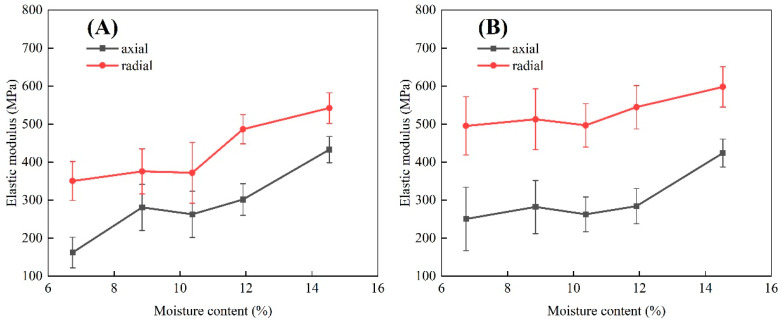

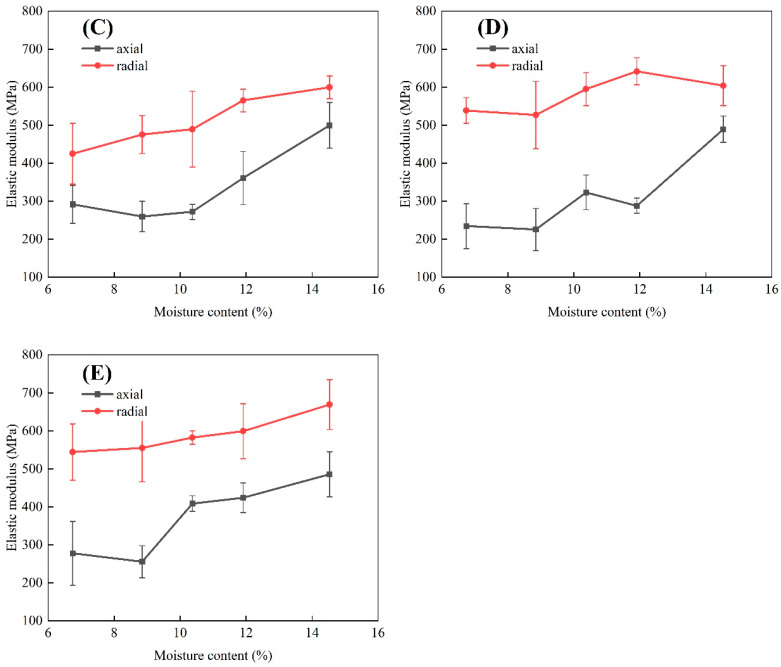
Relationship between elastic modulus and moisture content. (**A**–**E**) means aspect ratio is 1, 1.5, 2, 2.5, and 3, respectively.

The elastic modulus could be used to measure the difficulty of elastic deformation of the pellets, and its value was inversely proportional to the amount of elastic deformation under pressure. Under the condition of different aspect ratios, the relationship between elastic modulus and moisture content is shown in Figure 6. When the aspect ratio was 1 and 1.5, the axial and radial elastic modulus showed a trend of rising to decline and then rising. The optimum moisture content, aspect ratio, and axial elastic modulus were 6.74% d.b., 1, and 164.88 MPa, respectively. When the aspect ratios were 2, 2.5, and 3, the axial elastic modulus decreased and then increased, while the radial direction showed an upper trend. The optimum moisture content, aspect ratio, and axial elastic modulus were 8.85% d.b., 2.5, and 225.13 MPa, respectively. However, the elastic modulus was positively correlated with the moisture content, which was inconsistent with the conclusions obtained from peanut shells, potatoes, wheat, and other grains [30,31,32]. It was because gluten was mainly composed of elastic glutenin and extensible gliadin, whose combined action gave the gluten unique viscoelasticity. It was caused by the firmer binding strength of powder particles and the denser texture after being watered [33]. The radial elastic modulus was larger than the axial elastic modulus under the same moisture content, indicating that the radial compression could withstand less strain and be easier to break under the same stress. Moreover, in the compressive direction, there were fewer particles and inter-particle gaps in the radial direction than in the axial direction. Based on the test results, to prevent the crushing of the pellet in the design of packaging, transportation, and other process machinery, the radial compression elastic modulus of the pellet should be used as the design basis.

In order to predict the pellet elastic modulus, SPSS software (Version 25.0, International Business Machines Corporation, Armonk, NY, USA) was used to fit the functional relationship between the elastic modulus and the moisture content, the aspect ratio when the pellet was compressed in the axial and radial directions, as shown in Equations (8) and (9). The deterministic coefficient of both equations was 0.84, and the model fitted well.

(8) $$E_{a}=398.68-21.95X-49.82Y+2.16XY+8.63X^{2}+3.46Y^{2}\;R^{2}=0.84$$

(9) $$E_{r}=81.23+238.11X+8.88Y-4.35XY-29.07X^{2}+0.845Y^{2}\;R^{2}=0.84$$

where $E_{a}$ and $E_{r}$ are the axial and radial elastic modulus of the feed, respectively, MPa; *X* is aspect ratio; and *Y* is moisture content, %.

The feed moisture content should be less than 12.5% d.b. according to the industry standard [34]. It was substituted into the models, and the range of the elastic modulus of the pellet under the axial and radial compression could be obtained at 340.65–431.78 MPa and 478.64–622.21 MPa, respectively. Therefore, gluten pellets’ maximum allowable elastic modulus in actual production and transportation should be less than 340.65 MPa, and the optimal aspect ratio was 1.5.

### 3.3. Influence of Moisture Content and Aspect Ratio on Compressive Strength

**Figure 7:**
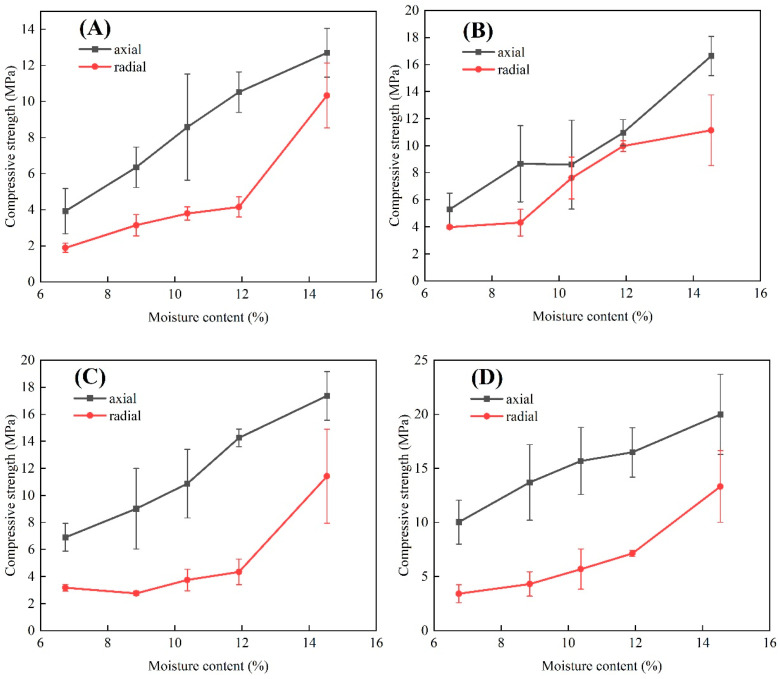

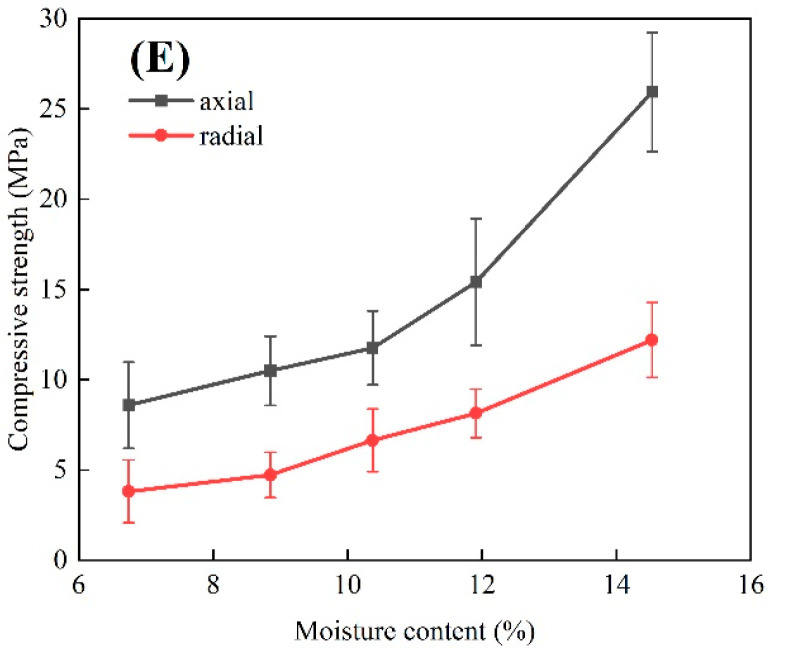
Relationship between compressive strength and moisture content. (**A**–**E**) means aspect ratio is 1, 1.5, 2, 2.5, and 3, respectively.

Figure 7 shows the relationship between compressive strength and moisture content of pellets under different aspect ratios and compressive directions. The axial and radial compressive strengths increased with the increase in moisture content under the same compressive direction. The compressive strength of radial compression was smaller than that of axial compression under the same moisture content. The compressive strength would increase significantly when the moisture content exceeds 11.91% d.b. The main reason was that from the view of processing, when the pellets were extruded in the ring die, the closer the powder in the die hole to the inner wall surface, the higher the flow velocity, and the material would form gradient stratification in the diameter direction, which would lead to shear damage under radial compression. From the moisture content perspective, the pellet’s internal binding force was different due to the different moisture content. Steam would be introduced to make the gluten gelatinized, and the cohesion increased before the pellet was formed. At the same time, water would condense in the space between the particles, and a liquid bridge would be formed at the contact point of the particles. When the moisture content was high, a liquid bridge would be formed between the powder particles at the contact point, and the strength would decrease [35].

On the contrary, when the moisture content of the pellet was low, the cohesion between particles would decrease, the strength of pellets would also decrease, and the ability to resist damage would decrease. Considering the compression area, under the same deformation amount of the pellet with the same moisture content, the larger the compression area, the higher the compressive strength and the stronger the ability to resist rupture. When the pellet was compressed radially, the indenter was in line contact with the pellet. The compression area was smaller than when the pellet was compressed in the axial direction. Therefore, the compressive strength in the radial direction was smaller than that in the axial compression. Based on the test results, in order to prevent the crushing of the pellet in the design of packaging, transportation, and other process machinery, the compressive strength of the pellet should be based on radial compression.

In order to predict the pellet compressive strength, SPSS software (Version 25.0, International Business Machines Corporation, USA) was used to fit the functional relationship between the compressive strength and the moisture content, the aspect ratio when the pellet was compressed in the axial and radial directions, as shown in Equations (10) and (11). The deterministic coefficient of both equations was 0.92 and 0.86, respectively, and the model fitted well.

(10) $${Pb}_{a}=2.58+2.72X-0.85Y+0.38XY-0.82X^{2}+0.07Y^{2}\;R^{2}=0.92$$

(11) $${Pb}_{r}=8.04+0.99X-1.89Y+0.08XY-0.24X^{2}+0.13Y^{2}\;R^{2}=0.86$$

where ${Pb}_{a}$ and ${Pb}_{r}$ are the axial and radial compressive strength of the feed, respectively, MPa; *X* is aspect ratio; and *Y* is moisture content, %.

According to the industry standard of pellet moisture content, the moisture content should be less than 12.5% d.b., and the maximum moisture content of the pellet was substituted into the models. The range of the compressive strengths of the pellet under the axial and radial compression could be obtained at 10.70–18.84 MPa and 6.25–9.31 MPa, respectively. Therefore, it was more likely to be crushed during radial compression, the minimum allowable compressive strength should be greater than 6.25 MPa, and the optimal aspect ratio of the pellet was 2.

### 3.4. Influence of Moisture Content and Aspect Ratio on Failure Energy

**Figure 8:**
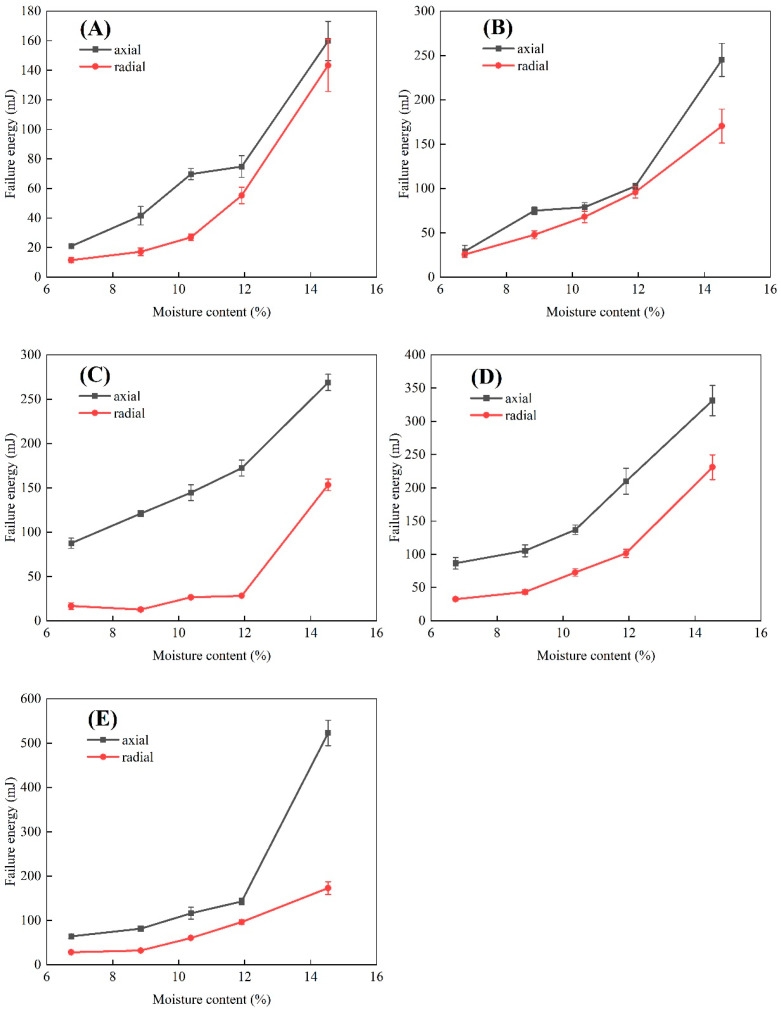
Relationship between failure energy and moisture content. (**A**–**E**) means aspect ratio is 1, 1.5, 2, 2.5 and 3, respectively.

The failure energy represented the minimum energy absorbed by the permanent failure. The larger the value, the more energy absorbed and the better the material’s toughness. Figure 8 shows pellet failure energy and moisture content change law under different aspect ratios and compressive directions. The failure energy of the pellet during axial and radial compression increased with the moisture content, regardless of the aspect ratio. The failure energy increased slowly when the moisture content was lower than 11.91% d.b. and increased rapidly when the moisture content was higher than 11.91% d.b. The radial compression was smaller than the axial compression failure energy under the same moisture content. The reason was that when the moisture content was high, the internal organization of the pellet would become soft, and it could withstand a large amount of deformation under pressure and absorb more energy, so the failure energy was enormous. While the pellet showed brittleness with a slight deformation under compression and little ability to absorb energy when the moisture content was low, it could cause less failure energy.

In order to predict the pellet failure energy, SPSS software (Version 25.0, International Business Machines Corporation, Armonk, NY, USA) was used to fit the functional relationship between the failure energy and the moisture content, the aspect ratio when the pellet was compressed in the axial and radial directions, as shown in Equations (12) and (13). The deterministic coefficient of both equations was 0.89, and the model fitted well.

(12) $$W_{a}=464.88-23.40X-101.68Y+16.55XY-22.94X^{2}+4.65Y^{2}\;R^{2}=0.89$$

(13) $$W_{r}=200.94+6.65X-50.46Y+2.34XY-4.35X^{2}+3.05Y^{2}\;R^{2}=0.89$$

where $W_{a}$ and $W_{r}$ are the axial and radial failure energy of the feed, respectively, MPa; *X* is aspect ratio; and *Y* is moisture content, %.

According to the industry standard of pellet moisture content, the moisture content should be less than 12.5% d.b. The range of the failure energy of the pellet under the axial and radial compression could be obtained at 107.92–251.48 mJ and 64.77–146.80 mJ, respectively. Therefore, the radial compression of the pellet should be avoided in the actual production and transportation, the minimum allowable failure energy should be greater than 64.77 mJ, and the optimum aspect ratio of the pellet was 2.

### 3.5. Simulation Results and Analysis

**Figure 9:**
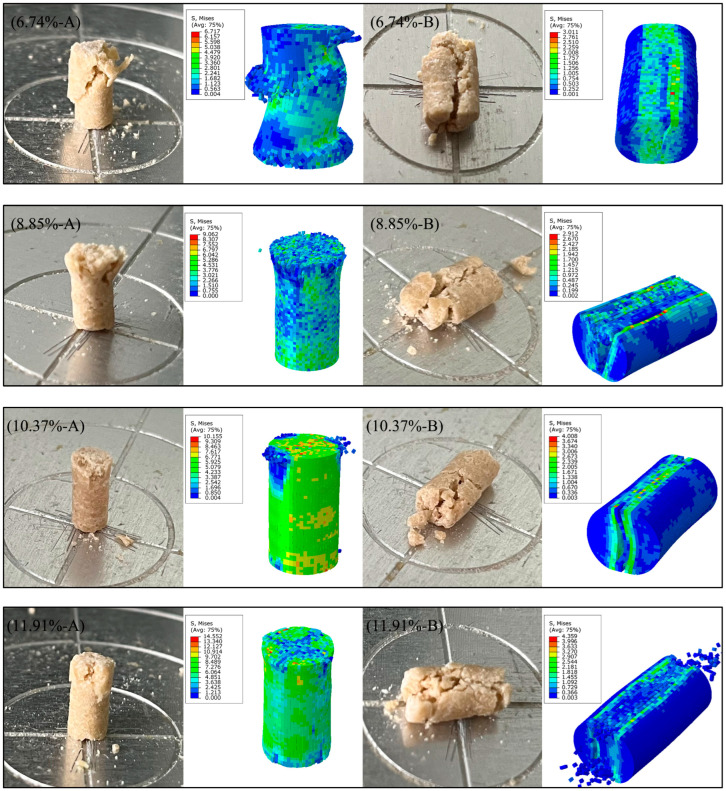

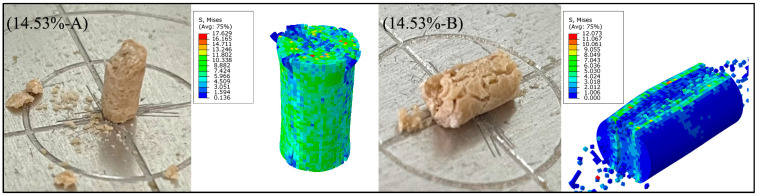
Real and simulated diagrams of feed compression fracture. The number 6.74%, 8.85%, 10.37%, 11.91%, and 14.53% before (**A**) or (**B**) means moisture content; (**A**,**B**) means compressive direction is axial and radial, respectively.

The internal stress distribution of pellets under compressive load was significant to studying its rupture form (the location and shape of the fracture cross-section). The fracture and simulated stress nephogram diagrams under axial and radial compression at different moisture contents are shown in Figure 9.

**Figure 10:**
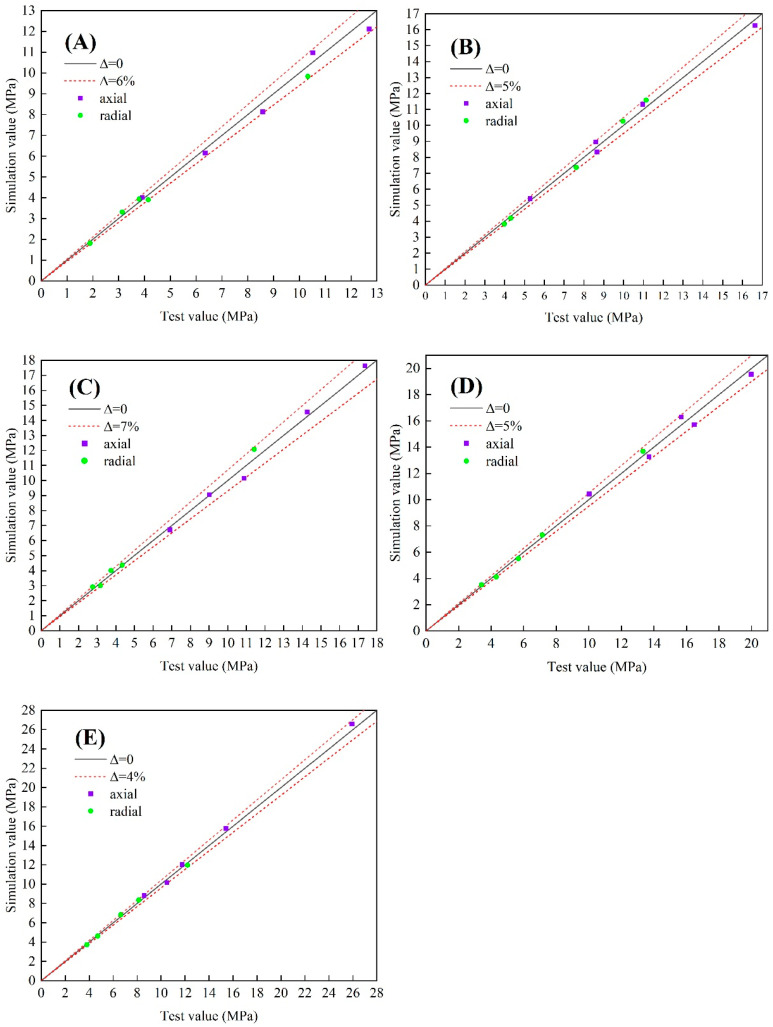
Error between test value and simulation value of failure stress. (**A**–**E**) means aspect ratio is 1, 1.5, 2, 2.5, and 3, respectively.

It could be seen from the broken pictures of the pellet compression test that under the condition of axial compression, the pellets with a moisture content of 6.74% d.b. were mainly broken along the 45° slope. The fracture mainly occurred in the pellet’s upper half along the compression surface’s periphery when the moisture content was higher than 6.74% d.b. The pellet contact surface was flattened, and fractures occurred along the axial and radial directions as the indenter dropped under radial compression conditions. It could be seen from the crushing stress nephogram of the pellet compression simulation that the axial and radial crushing forms of the pellets were consistent with the experimental results. Furthermore, the simulation accuracy of the maximum stress during rupture was analyzed. It could be seen from the simulation stress nephogram that when the aspect ratio was 2, the maximum rupture stress simulation value of each moisture content pellet was relatively close to the experimental value. The simulation and experimental values were calculated by Equation (8), which were within the range of 4–7%. The simulation accuracy was acceptable [36,37], as shown in Figure 10. It showed that using the finite element method to analyze the maximum stress of pellet compression fracture was feasible, and it was closely related to the moisture content. The errors may be the following: (1) Although the pellets selected by the author in the test have no defects on the outside, the internal situation is unknown. Defects such as cracks and tiny gaps may exist inside the actual pellets, while the geometric simulation models are meshing with the same size, and it is assumed that there are no defects and tiny gaps. The existence of defects will weaken the bonding force inside the pellets and reduce the stress required for failure, which will lead to errors between the stress value of pellet rupture and the simulated value. There are also errors in the direction of crack growth at the rupture. (2) The simulation input parameters of pellets, such as elastic modulus and density, all adopt the average value measured by pellets. However, there are differences between actual pellets, and the actual pellets may have a nonuniformity moisture content of individual pellets. However, the simulation pellets assume uniform moisture content, so that errors will occur between the simulation and test values. The simulated and experimental rupture stress values increase with the moisture content.

(14) $$∆=\left|\frac{T-S}{T}\right|\times 100\%$$

where ∆ is the relative error, %; T is the experimental value, MPa; and S is the simulation value, MPa.

## 4. Conclusions

(1) The elastic modulus, compressive strength, and failure energy of gluten pellets with different moisture content and aspect ratio under axial and radial compression were obtained through compression tests. The gluten pellet was more likely to be broken when radially compressed. The gluten pellets’ material property was anisotropic.
(2) The aspect ratio did not significantly affect the compressive strength. The elastic modulus, compressive strength, and failure energy of gluten pellets should be less than 340.65 MPa, 6.25 MPa, and 64.77 mJ, respectively, during manufacturing, packaging, and transportation.
(3) The established mechanical model with cohesive elements could be used to analyze the compression fracture stress of anisotropic property pellet feed. Moreover, the relative error between the experimental and simulation values was 4–7%.

## Figures and Tables

**Table 1:** Material parameters.

Pellet Feed									Cohesive Element			
MC (%) ^1^	E (MPa) ^2^			*μ* ^3^	G (MPa) ^4^			*ρ* (kg/m^3^) ^5^	BS ^6^	R (MPa/mm) ^7^	CFD (mm) ^8^	*ρ* (kg/m^3^) ^5^
	E1	E2	E3	Nu	G12	G13	G23					
6.74	424.70	424.70	290.28	0.40	176.96	176.96	103.67	1129.92	10.00	35.00	1.00 × 10^−3^	1.70 × 10^3^
8.85	475.25	475.25	259.41		169.73	169.73	92.65	1146.90				
10.37	489.11	489.11	272.01		174.68	174.68	97.15	1159.64				
11.91	565.07	565.07	360.65		201.81	201.81	128.80	1170.17				
14.53	599.48	599.48	499.39		214.10	214.10	178.35	1175.36				

^1^ MC: moisture content. ^2^ E: elastic modulus. ^3^ *μ*: Poisson’s ratio. ^4^ G: shear modulus. ^5^ *ρ*: density. ^6^ BS: bond strength. ^7^ R: rigidity. ^8^ CFD: critical failure displacement.

**Table 2:** Static compression test of pellet feed results.

Aspect Ratio	Moisture Content (%)	Elastic Modulus (MPa)		Compressive Strength (MPa)		Failure Energy (mJ)	
		Axial	Radial	Axial	Radial	Axial	Radial
1	6.74 ± 0.25	161.88 ± 40.52 ^a^	350.15 ± 51.29 ^a^	3.92 ± 1.25 ^a^	1.89 ± 0.26 ^a^	20.93 ± 1.33 ^a^	11.46 ± 1.74 ^a^
	8.85 ± 0.17	280.60 ± 60.42 ^ab^	375.38 ± 59.46 ^a^	6.35 ± 1.12 ^a^	3.14 ± 0.59 ^b^	41.51 ± 6.26 ^a^	17.10 ± 2.57 ^bc^
	10.37 ± 0.31	262.35 ± 61.26 ^ab^	371.83 ± 79.89 ^a^	8.58 ± 2.94 ^a^	3.79 ± 0.37 ^a^	69.74 ± 3.70 ^a^	26.91 ± 2.28 ^ab^
	11.91 ± 0.23	301.26 ± 42.07 ^b^	486.50 ± 38.68 ^a^	10.52 ± 1.12 ^a^	4.15 ± 0.56 ^b^	74.83 ± 7.37 ^a^	55.28 ± 5.55 ^c^
	14.53 ± 0.11	432.73 ± 34.51 ^c^	542.10 ± 40.23 ^a^	12.70 ± 1.36 ^b^	10.33 ± 1.80 ^c^	159.79 ± 13.28 ^b^	143.33 ± 17.73 ^d^
1.5	6.74 ± 0.25	250.36 ± 83.28 ^ab^	495.21 ± 76.61 ^a^	5.29 ± 1.20 ^ab^	3.98 ± 0.13 ^a^	29.07 ± 6.81 ^a^	25.43 ± 2.96 ^a^
	8.85 ± 0.17	281.84 ± 70.00 ^a^	512.43 ± 79.89 ^a^	8.66 ± 2.82 ^a^	4.31 ± 0.99 ^ab^	74.91 ± 4.16 ^a^	47.67 ± 4.47 ^a^
	10.37 ± 0.31	262.54 ± 45.49 ^c^	496.49 ± 57.29 ^a^	8.60 ± 3.29 ^bc^	7.61 ± 1.55 ^ab^	78.87 ± 4.99 ^ab^	67.99 ± 6.49 ^a^
	11.91 ± 0.23	283.97 ± 46.54 ^a^	544.41 ± 57.21 ^a^	10.96 ± 0.97 ^a^	9.97 ± 0.39 ^b^	102.55 ± 3.29 ^a^	95.74 ± 6.45 ^a^
	14.53 ± 0.11	423.76 ± 36.82 ^ab^	597.73 ± 53.11 ^a^	16.63 ± 1.45 ^c^	11.14 ± 2.61 ^c^	245.02 ± 18.63 ^b^	170.37 ± 19.16 ^b^
2	6.74 ± 0.25	290.28 ± 50.69 ^a^	424.70 ± 80.65 ^a^	6.90 ± 1.02 ^a^	3.18 ± 0.25 ^bc^	87.51 ± 5.81 ^a^	16.61 ± 3.59 ^bc^
	8.85 ± 0.17	259.41 ± 40.09 ^a^	475.25 ± 51.67 ^a^	9.01 ± 2.98 ^a^	2.76 ± 0.17 ^a^	120.88 ± 3.58 ^a^	12.63 ± 1.87 ^a^
	10.37 ± 0.31	272.01 ± 21.63 ^a^	489.11 ± 99.65 ^a^	10.87 ± 2.54 ^a^	3.75 ± 0.80 ^bc^	144.56 ± 8.86 ^a^	26.46 ± 1.50 ^bc^
	11.91 ± 0.23	360.65 ± 69.63 ^ab^	565.07 ± 31.95 ^a^	14.26 ± 0.65 ^a^	4.34 ± 0.95 ^bc^	172.24 ± 9.03 ^a^	28.11 ± 1.36 ^bc^
	14.53 ± 0.11	499.39 ± 59.56 ^b^	599.48 ± 28.53 ^a^	17.36 ± 1.80 ^b^	11.42 ± 3.47 ^c^	268.87 ± 19.32 ^b^	153.27 ± 16.59 ^b^
2.5	6.74 ± 0.25	234.25 ± 59.24 ^a^	538.40 ± 33.44 ^a^	10.03 ± 2.03 ^a^	3.41 ± 0.83 ^a^	86.49 ± 8.69 ^a^	32.47 ± 2.68 ^ab^
	8.85 ± 0.17	225.13 ± 55.56 ^a^	526.65 ± 88.87 ^a^	13.70 ± 3.49 ^a^	4.31 ± 1.13 ^a^	105.22 ± 9.02 ^a^	43.24 ± 3.42 ^a^
	10.37 ± 0.31	322.91 ± 45.60 ^a^	594.90 ± 43.48 ^a^	15.68 ± 3.10 ^a^	5.68 ± 1.86 ^a^	136.83 ± 7.13 ^a^	72.65 ± 5.66 ^ab^
	11.91 ± 0.23	287.66 ± 20.14 ^a^	641.58 ± 35.60 ^a^	16.48 ± 2.28 ^a^	7.14 ± 0.29 ^a^	209.75 ± 19.32 ^a^	101.38 ± 6.27 ^ab^
	14.53 ± 0.11	489.21 ± 34.60 ^b^	603.95 ± 52.56 ^a^	19.98 ± 3.71 ^b^	13.33 ± 3.33 ^a^	331.22 ± 22.75 ^b^	230.96 ± 18.51 ^b^
3	6.74 ± 0.25	277.60 ± 84.04 ^ab^	544.18 ± 74.11 ^ab^	8.59 ± 2.39 ^a^	3.81 ± 1.74 ^a^	63.90 ± 4.88 ^a^	27.96 ± 1.10 ^a^
	8.85 ± 0.17	255.24 ± 42.31 ^ab^	554.82 ± 89.21 ^ab^	10.49 ± 1.91 ^a^	4.72 ± 1.26 ^a^	81.10 ± 2.42 ^a^	31.86 ± 2.25 ^a^
	10.37 ± 0.31	408.23 ± 20.51 ^bc^	582.13 ± 17.80 ^ab^	11.76 ± 2.04 ^a^	6.64 ± 1.73 ^a^	116.04 ± 13.28 ^a^	60.19 ± 2.34 ^a^
	11.91 ± 0.23	423.65 ± 38.87 ^a^	598.89 ± 72.13 ^a^	15.41 ± 3.50 ^a^	8.14 ± 1.34 ^a^	142.65 ± 7.13 ^a^	96.06 ± 5.64 ^a^
	14.53 ± 0.11	485.50 ± 59.29 ^c^	669.12 ± 65.48 ^b^	25.93 ± 3.30 ^b^	12.20 ± 2.07 ^b^	522.82 ± 28.78 ^b^	172.79 ± 14.56 ^b^

The values are presented as means ± SD. The same aspect ratio in the same column with different lowercase letters means a significant difference (*p* < 0.05).

**Table 3:** Independence test of axial and radial elastic modulus.

Aspect Ratio	Elastic Modulus (MPa)		*t*-Value	*p*-Value
	Axial	Radial		
1	287.76 ± 97.14	425.19 ± 84.24	−2.390	0.044
1.5	300.49 ± 70.30	529.25 ± 43.11	−6.203	0.000
2	336.55 ± 99.07	510.72 ± 70.64	−3.201	0.013
2.5	311.83 ± 106.90	581.10 ± 47.85	−5.141	0.001
3	370.04 ± 99.23	589.83 ± 49.34	−4.435	0.002

The values are presented as means ± SD.

**Table 4:** Significance analysis.

Source of Variance	Elastic Modulus (MPa)		Compressive Strength (MPa)		Failure Energy (mJ)	
	F-Value	*p*-Value	F-Value	*p*-Value	F-Value	*p*-Value
Moisture content	10.967	<0.001	41.557	<0.001	48.105	<0.001
Aspect ratio	3.505	0.240	6.749	<0.001	6.138	0.147
Compressive direction	171.961	<0.001	121.716	<0.001	57.293	<0.001

## Data Availability

The data presented in this study are available on request from the corresponding author. The data are not publicly available due to manufacturer’s requirement.
